# Regulation of root patterns in mammalian teeth

**DOI:** 10.1038/s41598-017-12745-1

**Published:** 2017-10-05

**Authors:** Hyejin Seo, Jinsun Kim, Jae Joon Hwang, Ho-Gul Jeong, Sang-Sun Han, Wonse Park, Kanghyun Ryu, Hong Seomun, Jae-Young Kim, Eui-Sic Cho, Joo-Cheol Park, Kyung-Seok Hu, Hee-Jin Kim, Dong-Hyun Kim, Sung-Won Cho

**Affiliations:** 1 Division of Anatomy and Developmental Biology, Department of Oral Biology, Yonsei University College of Dentistry, Seoul, Korea; 2 Brain Korea 21 Plus Project, Yonsei University College of Dentistry, Seoul, Korea; 3 Department of Oral and Maxillofacial Radiology, Yonsei University College of Dentistry, Seoul, Korea; 4 Department of General Dentistry, Yonsei University College of Dentistry, Seoul, Korea; 5 Department of Electrical and Electronic Engineering, Yonsei University, Seoul, Korea; 6 National Institute of Biological Resources, Incheon, Korea; 7 Department of Biochemistry, School of Dentistry, IHBR, Kyungpook National University, Daegu, Korea; 8 Cluster for Craniofacial Development and Regeneration Research, Institute of Oral Biosciences, School of Dentistry, Chonbuk National University, Jeonju, Korea; 9 Department of Oral Histology-Developmental Biology and Dental Research Institute, School of Dentistry, Seoul National University, Seoul, Korea

## Abstract

Mammalian teeth have diverse pattern of the crown and root. The patterning mechanism of the root position and number is relatively unknown compared to that of the crown. The root number does not always match to the cusp number, which has prevented the complete understanding of root patterning. In the present study, to elucidate the mechanism of root pattern formation, we examined (1) the pattern of cervical tongues, which are tongue-like epithelial processes extending from cervical loops, (2) factors influencing the cervical tongue pattern and (3) the relationship among patterns of cusp, cervical tongue and root in multi-rooted teeth. We found a simple mechanism of cervical tongue formation in which the lateral growth of dental mesenchyme in the cuspal region pushes the cervical loop outward, and the cervical tongue appears in the intercuspal region subsequently. In contrast, when lateral growth was physically inhibited, cervical tongue formation was suppressed. Furthermore, by building simple formulas to predict the maximum number of cervical tongues and roots based on the cusp pattern, we demonstrated a positive relationship among cusp, cervical tongue and root numbers. These results suggest that the cusp pattern and the lateral growth of cusps are important in the regulation of the root pattern.

## Introduction

Tooth development initiates from the thickening of epithelium, and the tooth germ continues to form its shape through the lamina, bud, cap and bell stages. Crown morphogenesis is followed by cell differentiation and mineralization, and root formation and eruption takes place^1^. Most teeth in fish, amphibians and reptiles are haplodont with one cusp and one root, while the posterior teeth of mammals, including premolars and molars, show a variety of crown shapes with species-specific patterns in the crown and root. Minor variations of the tooth crowns and roots are the characters most amenable to evolutionary change^2^.

The crown has been used as a key morphological feature for species identification in animals due to its advantages of accessibility and diversity. The pattern in the crown and root of posterior teeth varies among different species of animals but is generally consistent within the same species. The crown is also widely used as a tool for establishing the developmental principles that link genotype to phenotype^3,4^. However, root morphogenesis has not been studied in depth due to its difficult accessibility compared to that of the crown, since roots are covered in alveolar bone and surrounding tissues. Roots can only be observed by x-ray images or tooth extraction.

The crown has been the main focus in all studies dealing with the evolution of extant and extinct species, but it is still difficult to distinguish one genus from the other genus due to their similar crown pattern in molars. Root pattern was also demonstrated to be characteristics equivalent to those of the crown in murine rodent evolutionary studies^5^. However, it is also not efficient to solely use the root pattern for species identification. For example, teeth with different numbers of cusps often have the same number of roots; the premolars in carnivores, the human mandibular first molar and the mouse mandibular first molar have one, five and seven cusps, respectively, but all these teeth have only two roots. On the other hand, teeth with same numbers of cusps occasionally have the different numbers of roots. Therefore, the combination of the root pattern and the cusp pattern would substantially improve species identification.

Functionally, the crown and root are tightly connected, as the mastication is not possible without the crown or root. The root anchors teeth to the alveolar bone and transmit occlusal forces from the crown to the bone. Root morphogenesis has been thought to be closely related to crown morphogenesis, considering that root development always follows crown development in all teeth. A report found that supernumerary cusps in a tooth are frequently associated with supernumerary roots^6^, suggesting the dependence of the root pattern on the cusp pattern. Other studies have also described the root pattern in association with crown patterns, such as the number of cusps, the location of cusps and the crown outline in maxillary molars, but not in mandibular molars^7,8^. In addition, a comparative study of molar morphology in extant mammals suggested a positive correlation between the root number and cusp number in the maxilla^3^. Nonetheless, no study has shown the mechanism by which the crown pattern influences the root pattern in multi-rooted teeth.

In the present study, in order to investigate the association of root pattern and crown pattern and to elucidate the mechanism of formation of root pattern, the pattern changes of cervical tongues in mouse and rat tooth germs were examined and compared from the cap stage, in which crown morphogenesis is still in progress. The cervical tongue named in this study, also known as a tongue-like extension^9^, is a tongue-shaped epithelial process extending from cervical loop. Elongation and the contact of these cervical tongues form discrete regions, resulting in the furcation zone of roots^9^. Therefore, it has been suggested that the pattern of cervical tongues plays an important role in the determination of the numbers, lengths, and shapes of the roots^10–13^.

We found that cervical tongues were observed from the early bell stage. By modulating the degree of lateral growth of the mouse tooth germ *in vitro*, we found that lateral growth is important in the appearance of cervical tongues. Lateral growth refers to the growth of tooth in buccal, lingual, mesial, and/or distal direction in this study. We also presented formulas showing a positive relationship between cusp number and cervical tongue number. Furthermore, various cusp patterns and root patterns observed in actual mammalian teeth were simulated by a simple mechanism in which the lateral growth of dental mesenchyme pushes the cervical loop outward, and in turn, the cervical tongue appears in the intercuspal region.

## Results

### Cervical tongues are present from the bell stage in mouse and rat first molars

**Figure 1:**
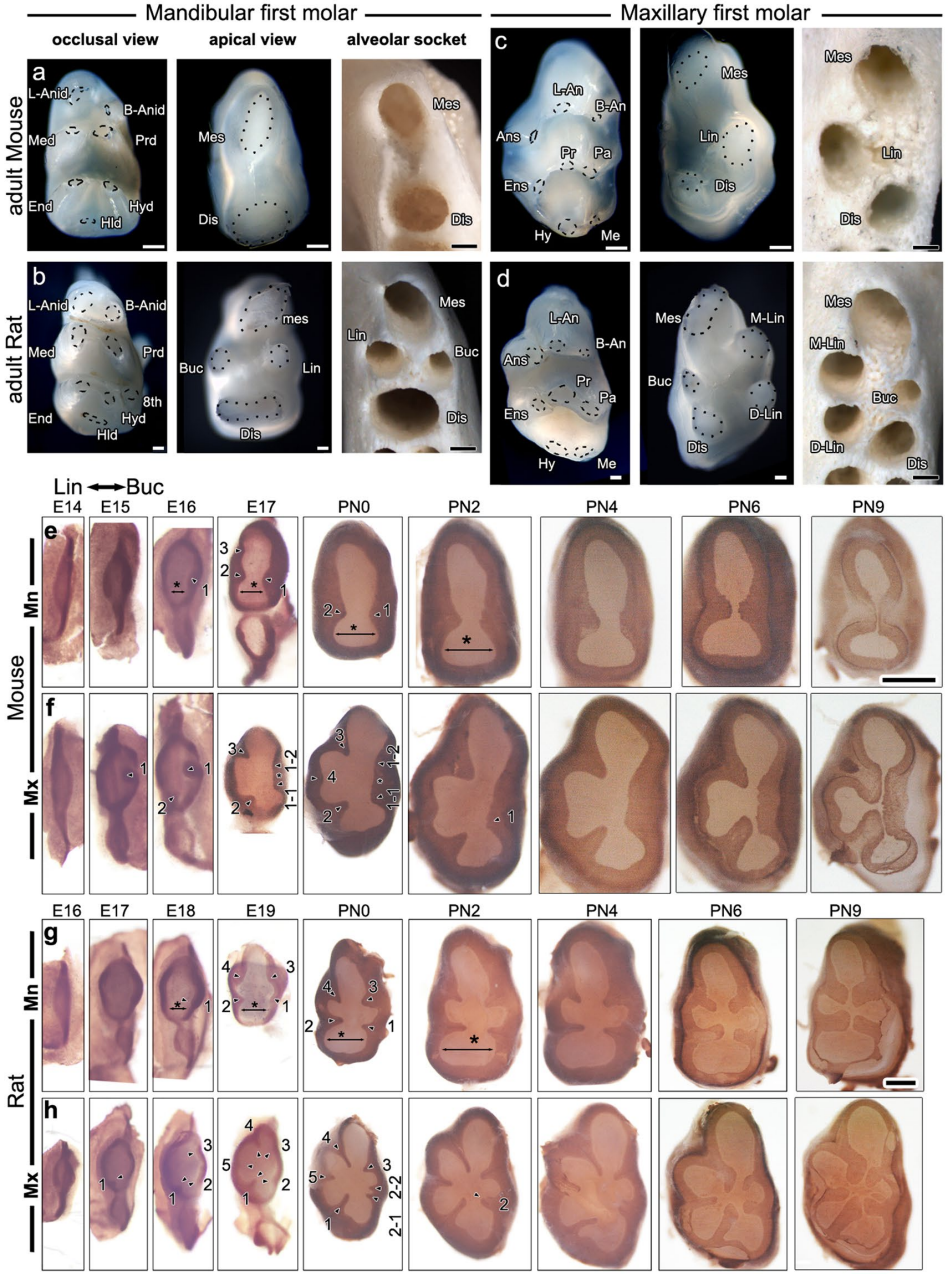
Cervical tongues determine the root pattern. **(a**–**d**) Cusp (occlusal view) and root pattern (apical view) in first molars of mandible and maxilla from PN (postnatal day)14 mouse (**a**,**b**) and PN21 rat (**c**,**d**). Alveolar sockets reveal the number of roots which concurrent with the number of roots depicted in apical view. Notice that mouse and rat share the same number of cusp in their first molars but their root numbers are different. **(e**,**f)** Localization of E-Cadherin protein in the dental epithelium of whole-mount first molars of mouse E (embryonic day)14, 15, 16 and 17; and PN0, 2, 4, 6 and 9 visualizes cervical tongues (arrowheads) from the apical view. Two and three well-developed cervical tongues are formed in mouse mandibular and maxillary first molar, respectively (arrowheads in **e** and **f**). Buccal cervical tongues at E17 and PN0 merged together to form single well-developed cervical tongue at PN4 in maxilla (1–1, 1–2 and 1 in **f**). **(g**,**h)** Localization of E-Cadherin protein in the dental epithelium of whole-mount first molars of rat E16, 17, 18 and 19; and PN0, 2, 4, 6 and 9 visualizes cervical tongues (arrowheads) from the apical view. Four and five well-developed cervical tongues are formed in rat mandibular and maxillary first molar, respectively (arrowheads). Notice doublet of buccal cervical tongues in maxilla at PN0 form single cervical tongue at PN2 (2–1, 2–2 and 2 in **h**). Notice that the distance between buccal and lingual cervical loops in cuspal area increase from E16 to PN4 and decreased from PN6 (asterisks in **e** and **g**). B-anid: buccal anteroconid, L-anid: lingual anteroconid, Prd: protoconid, Med: metaconid, Hyd: hypoconid, End: entoconid, Hld: hypoconulid. For maxilla, B-An: buccal anterocone, L-An: lingual anterocone, Ans: anterostyle, Pa: paracone, Pr: protocone, Ens: enterostyle, Me: metacone, Hy: hypocone. Scale bar: **a**–**h**, 500 µm.

*Mus musculus* (common name: house mouse) and *Rattus norvegicus* (common name: brown rat) in family Muridae exhibit similar molar crown shape with two rows of cusps in the mandible and three rows of cusps in the maxilla (Fig. 1a–d). The two different species have molars with seven cusps in the mandible and eight cusps in the maxilla. Interestingly, mice and rats have two and four roots in the mandible and three and five roots in the maxilla, respectively. The root numbers were confirmed by the respective alveolar sockets. Therefore, crown and root patterning of first molars during tooth development were compared between mouse and rat.

To investigate the root pattern of first molar in these two species, the developmental pattern of cervical tongues was tracked from cap stage to the stage when cervical tongues made contact with each other (Fig. 1e–h). E-cadherin, which is localized only in the epithelium and practically defines the boundary between the epithelium and the mesenchyme, was detected in the dental epithelium of mandibular and maxillary first molar tooth germs from the cap stage, mouse embryonic day (E) 14 and rat E16, and thereafter to visualize cervical tongues. Cervical tongues were observable in the maxilla from mouse E15 and rat E17 (Fig. 1f,h) and in the mandible from mouse E16 and rat E18 (Fig. 1e,g) at corresponding regions where evident cervical tongues were present at postnatal day (PN) 0 both in mice and rats (Fig. 1e–h). Strikingly, the cervical tongues were present from the bell stage, which is the stage before crown morphogenesis had completed and HERS had formed.

### Pattern of cervical tongues is different between mouse and rat first molars

The cervical tongues were observed in between cusps located in the axial outline of the tooth but not in all intercuspal regions. This was a common phenomenon for both mouse and rat first molars (Fig. 1e–h). However, the number of well-developed cervical tongues that meet each other and practically function to determine the root pattern was different between the mouse and rat. In mandibular first molars of mice and rats at PN6, two and four well-developed cervical tongues were observed to create two and four discrete regions, respectively, concurring with the future number of roots. Interestingly, a lingual cervical tongue observed between the lingual anteroconid and metanocid at E17 in mice gradually shrank and disappeared at PN0 (Fig. 1e). In maxillary first molars, three cervical tongues in the maxillary first molar of mice and five cervical tongues in rats at PN6 almost fused at PN9 (Fig. 1f,h). Although the mouse maxillary first molar has five cervical tongues at PN0, two buccal cervical tongues did not develop independently and merged into a wide buccal cervical tongue, and the lingual cervical tongue located between the anterostyle and enterostyle did not sufficiently develop to meet the other buccal or lingual cervical tongue (Fig. 1f). Notably, the maxillary first molar of rats occasionally developed a doublet of cervical tongues between the paracone and metacone at PN0, but these two cervical tongues fused together to form a single well-developed cervical tongue later at PN2 or PN4 (Fig. 1h). The distance between buccal and lingual cervical loops in cuspal area increased from E16 to PN4 (Fig. 1e,g).

The position and the number of cervical tongues were further confirmed by *Ptch2* expression. *Ptch2* mRNA expression in the apical end of the inner dental epithelium revealed the cervical tongues by distinguishing the epithelial-mesenchymal border from the apical view (Fig. S1).

### Crown pattern was related to the pattern of cervical tongues

To identify the characteristics of the crown pattern that contribute to the pattern of cervical tongues, the crown morphology of the non-erupted mandibular and maxillary first molars extracted from PN14 mice and PN21 rats was compared. Because cervical tongues were generally observed in the intercuspal region, the intercuspal distance (in ratio to the mesiodistal diameter of the first molar in each species) and axial outline concavity were considered as crown morphological features for the pattern formation of cervical tongues.

**Figure 2:**
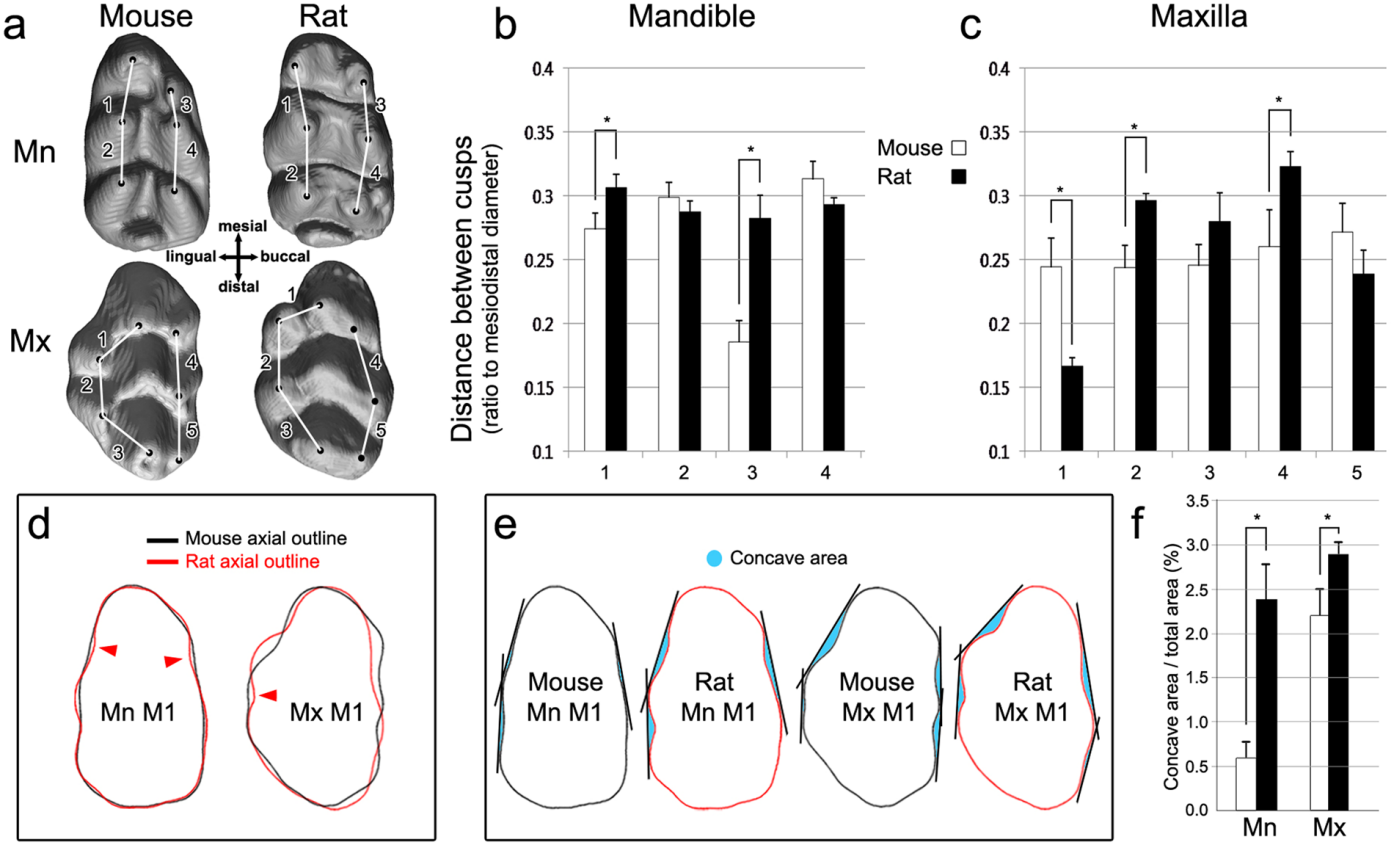
Relationship of intercuspal distance and concavity of axial outline with cervical tongue pattern. (**a**) Three-dimensionally (3D) reconstructed micro-computed topographies of PN14 mouse and PN21 rat first molars (M1) scaled to the same size from occlusal view. Black dots indicate highest points of each cusps and the lines connecting them are the reference lines for distance between cusps measured. **(b**,**c)** The distance between cusps in ratio to the mesiodistal diameter are compared between mouse (white bar) and rat (filled bar) in mandible (Mn) and maxilla (Mx). **(d)** Two-dimensional average (median) crown outlines of PN14 mouse and PN21 rat first molars (n = 5) generated based on Radial Fourier method is depicted. Concave axial outline in intercuspal regions where distinctions are observed between mouse outlines (black line) and rat outlines (red lines) are indicated by black arrowheads. **(e)** For the degree of concavity, tangent lines touching two convex outlines next to an intercuspal region were created in each tooth, and the area between the tooth outline and the tangent line was obtained and colored in light blue. **(f)** The concave area was measured and scaled to the total tooth area of two-dimentional outline, and then compared between mouse and rat (n = 5). *P* < 0.05.

The distance between cusps was greater in rats compared to mice in the intercuspal regions between the lingual anteroconid–metaconid, buccal anteroconid–protoconid, anterostyle–entostyle and buccal anterocone–paracone, where only rats had well-developed cervical tongues in the first molar (Fig. 2a,b). This result suggests the positive relationship between intercuspal distance and cervical tongue formation. Interestingly, the intercuspal distance between lingual anterocone and anterostyle, where both mice and rats have well-developed cervical tongues, was significantly greater in mice compared to rats (Fig. 2c). This intercuspal region had the shortest intercuspal length but showed the highest concavity of the axial outline in both mice and rats (Fig. 2c), which suggests that the concavity of axial outline is another factor for the pattern formation of cervical tongues.

In both species, the axial outline in the intercuspal regions was generally concave, especially where cervical tongues were formed. Intercuspal regions facing a diastema or second molars were exceptional in that cervical tongues were not observed in these regions, including between the buccal anteroconid–lingual anteroconid, entoconid–hypoconulid, hypoconid–hypoconulid, anterocone–anterostyle and metacone–hypocone. An intriguing difference was also found between the axial outlines of the maxillary and mandibular first molars of mice and rats. The intercuspal regions where only rats had well-developed cervical tongues showed higher concavity in rats compared to mice (Fig. 2d). The total concave area between the tooth outline and the tangent line relative to the total tooth area was larger in rats than in mice (Fig. 2e,f). The intercuspal distance and axial outline concavity were greater in rats than in mice, which might increase the appearance and development of cervical tongues.

### Cervical tongue in an intercuspal region is a passive product of mesenchymal cell proliferation in the dental papilla of the cuspal region

**Figure 3:**
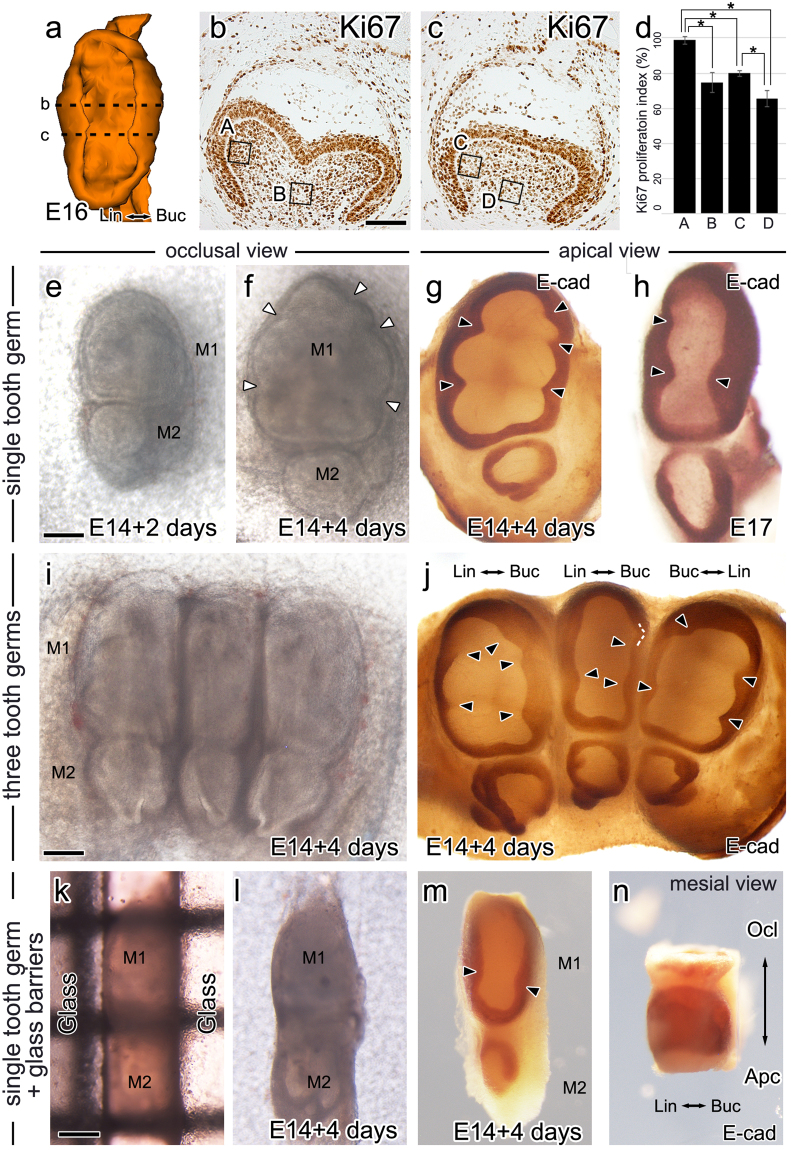
Alteration in cervical tongue pattern by the lateral growth and the growth inhibition. (**a**) Epithelium of bell stage (E16) tooth germ was 3-D reconstructed from serial sections and is viewed from apical side. Dashed lines indicate frontal section level of cusps and of cervical tongue, which were depicted in panel b and c, respectively. **(b**,**c)** The localization of Ki67 protein, a nuclear marker for cell proliferation, is observed in most of inner dental epithelial cells at the level of cusps (**b**) and of cervical tongue (**c**). The total number of cells and the number of Ki67-positive cells were counted in the 50 µm * 50 µm square in the center of dental papilla (B, D) and in the lateral side of dental papilla (A, C). (**d**) The Ki67 proliferation index shows the significantly higher value in lateral side of dental papilla at the level of cusp than other area. **(e)** Single tooth germ cultured *in vitro* for 2 days from E14. **(f**,**g)** Single tooth germ cultured *in vitro* for 4 days from E14. From occlusal view, concave axial outlines can be observed in between each intercuspal region (white arrowheads). From apical view, whole-mount E-cadherin immunostaining reveals development of five sharp cervical tongues in tooth germ cultured *in vitro* (black arrowheads). **(h)** Three cervical tongues are observed *in vivo* E17 tooth germ (black arrowheads) from apical view. **(i**,**j)** Three E14 tooth germs were placed side by side and cultured for 4 days to mimic the *in vivo*-like lateral growth inhibition. After 4 days, from occlusal view, tooth germs, particularly the central tooth germ, show flattened outline where pressure was applied. E-cadherin immunostaining of tooth germs showed short and blunt three cervical tongues in central tooth germ. No cervical tongue is observed in between the buccal and lingual anteroconids, though the mesial side of the central tooth germ does not contact with other tooth germs. The surface of buccal anteroconid is contact with surface of adjacent tooth germ (white dotted line). **(k**–**n)** An E14 tooth germ is placed in between two glass barriers from occlusal view and cultured for 4 days (**k** and **l**). After 4 days, tooth germ shows flattened outline where pressure was applied by glass barriers. E-cadherin immunostaining of tooth germs shows short and blunt three cervical tongues from apical view. From mesial view, the buccal and lingual sides of tooth germ are observed flat. Scale bars: **b**,**c**, 100 µm and **e**–**n**, 200 µm. *P* < 0.05. Lin: Lingual, Buc: Buccal, Ocl: Occlusal, Apc: Apical.

To determine why cervical tongues appeared at specific positions in the cervical loop, the localization of the Ki67 protein, a nuclear marker for cell proliferation, in developing mouse teeth was examined at E16. Most cells in the dental epithelium and dental mesenchyme were positive for Ki67 (Fig. 3b,c). When cell proliferation was compared between the sections at the level of the cusps (Fig. 3b) and at the level of the cervical tongues (Fig. 3c), a difference was observed in the dental papilla but not in the dental epithelium. There was no apparent difference in epithelial cell proliferation between cervical tongues and the neighboring epithelium. The lateral side of the dental papilla at the level of the cusps showed a remarkably higher proliferation index than the lateral side of the dental papilla at the level of the cervical tongue and the center regions of the dental papilla (Fig. 3d). Additionally, the distance between buccal and lingual cervical loops in the cuspal area of all first molars increased from E16 to PN4 (Fig. 1e,g). These results suggest that the condensed population of proliferating dental mesenchymal cells push the dental epithelium and cervical loop laterally in the cuspal region of tooth germs at the bell stage, and then the residual dental epithelium in the intercuspal regions may passively appear as a cervical tongue.

### Inhibition on the lateral growth of tooth germ decreases the number of cervical tongues

An *in vitro* culture system was utilized to confirm whether surrounding tissue has an influence on the axial outline and the cervical tongue pattern. Compared to *in vivo* tooth germs, tooth germs cultured *in vitro* had less surrounding tissue and therefore less inhibition on the lateral growth. When mouse mandibular E14 tooth germs were cultured for 4 days *in vitro*, tooth germs were rounder and showed a higher ratio of buccolingual diameter to mesiodistal diameter than *in vivo* E17 tooth germs (Fig. 3e–h). The axial outline was more convex in the cuspal region and more concave in the intercuspal region compared to E17 tooth germs from the occlusal view (Fig. 3g,h). Unlike E17 tooth germs, tooth germs cultured *in vitro* presented five cervical tongues, each of which was located in every intercuspal region except for the intercuspal regions next to the hypoconulid where it abuts the second molar (Fig. 3f,g). The tip of the cervical tongue was sharper in shape in tooth germs cultured *in vitro* than the E17 tooth germs. Intriguingly, a cervical tongue between two anteroconids was observed in the cultured tooth germ, where a cervical tongue has never been observed *in vivo*, even in mouse and rat mandibular first molars (Fig. 1e–h).

To confirm whether the surrounding tissue influences the pattern of cervical tongues, *in vivo*-like inhibition of lateral growth was created *in vitro* by placing three mouse mandibular tooth germs at E14 side by side and culturing them for 4 days (Fig. 3i,j). Tooth germs placed at either end would exhibit lateral growth inhibition on the buccal side based on our experimental design, while the tooth germ placed in the center would exhibit lateral growth inhibition on both the buccal and lingual sides. After 4 days, the buccolingual diameter of the tooth germ and the number of cervical tongues were smaller in the central tooth germ compared to tooth germs placed at either end. Tooth germs were obtained with a more flat outline where they contacted other tooth germs compared to the solely cultured tooth germs (Fig. 3f,i). Interestingly, the length and the tip of the cervical tongues were shorter and blunter, respectively, in the sides in contact with other tooth germs. The changes in the axial outline and cervical tongue pattern where it was in contact with other tooth germs may have resulted from the physical and molecular effects between the tooth germs next to each other. Therefore, in order to mimic the physical inhibition of lateral growth from the surrounding and to exclude any molecular effects, we placed an E14 tooth germ between two glass barriers and cultured it *in vitro*. After 4 days we obtained tooth germs with flattened buccal and lingual outline from occlusal view, similar with the central tooth germ in triplet culture (Fig. 3l). The cervical tongue pattern of the tooth germs cultured with glass barriers was similar to those of the central tooth germ in triplet culture and the *in vivo* E17 tooth germ (Fig. 3h,m). The buccal and lingual sides of tooth germ were observed to be flat from mesial view as well (Fig. 3n). These results indicate that the physical inhibition of the buccolingual growth of tooth germ suppresses the formation and development of cervical tongues. Interestingly, no cervical tongue was observed between the buccal and lingual anteroconids of central tooth germ in triplet culture and tooth germ between glass barriers (Figs 3j and S2c), although the mesial side of the central tooth germ did not contact with other tooth germs. The less the buccal anteroconid contacted with adjacent tooth germ, the longer and sharper the cervical tongue developed between the buccal and lingual anteroconids (Fig. S2d,e). These results suggest a possibility that cervical tongue in the intercuspal region is a passive product of lateral growth in the cuspal region.

### Root number can be estimated by simple formulas based on cusp number

**Figure 4:**
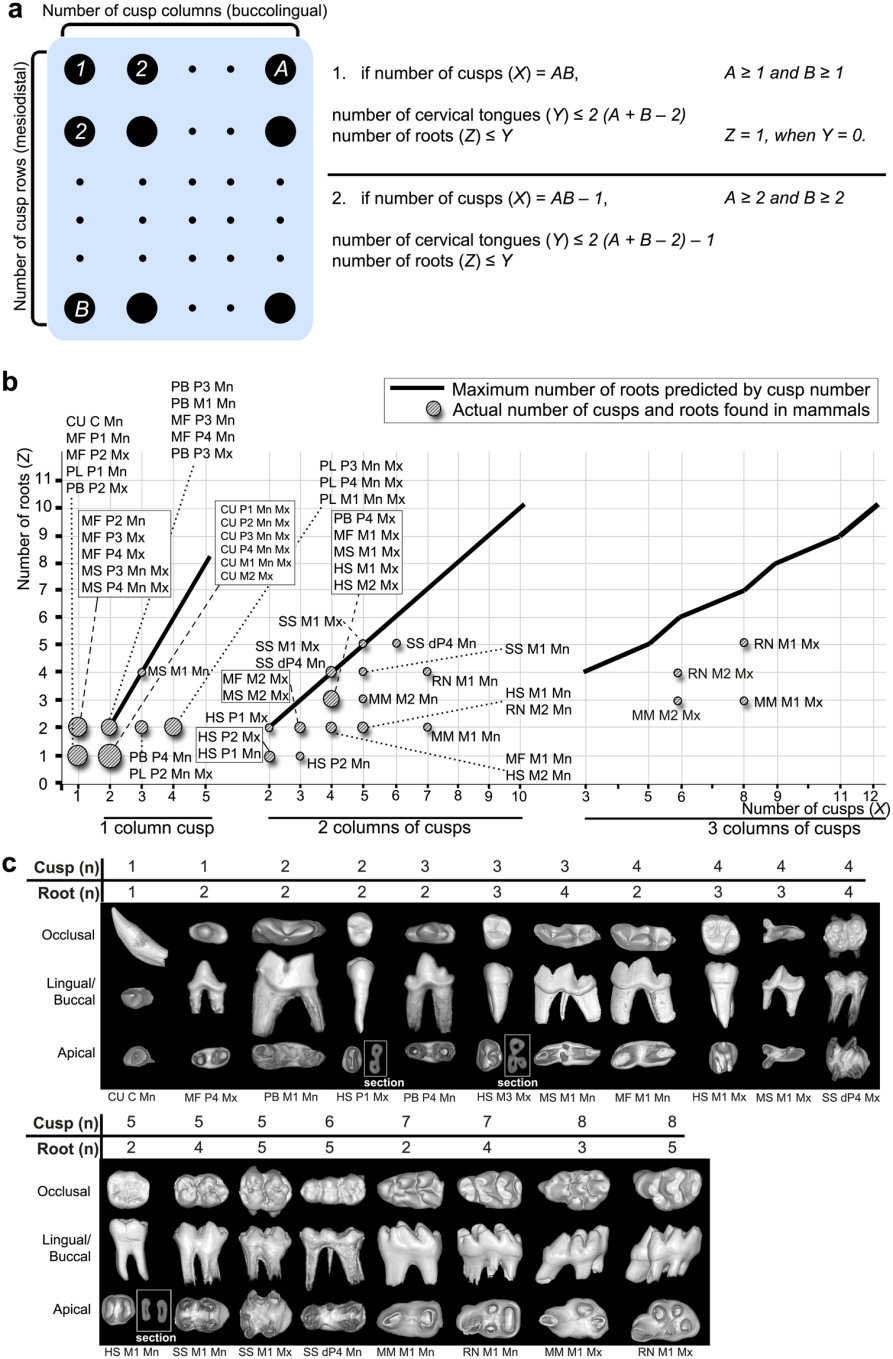
Positive relationship among the cusps, the cervical tongues and the roots. **(a**) A schematic diagram and formulas to predict maximum number of cervical tongues (*Y*) and roots (*Z*) depending on the cusp arrangement and cusp number (*X*) in mammalian teeth. The variety of cusp patterns is organized in columns (*A*) and rows (*B*). **(b)** Each of three graphs based on the formulas show a positive relationship between the estimated maximum number of roots and the cusp number. There is a strict linear relationship between cusp number and maximum root number in teeth where the number of cusp columns is one or two. All the actual numbers of roots are the same or less than the estimated maximum number of roots. **(c)** Lingual or buccal, occlusal and apical view of the crown and root and their numbers in various mammalian premolars and molars. HS: *Homo sapiens*, MF: *Martes flavigula*, MS: *Mustela sibirica*, PL: *Phoca largha*, PB: *Prionailurus bengalensis*, MM: *Mus musculus*, CU: *Callorhinus ursinus*, RN: *Rattus norvegicus*, SS: *Sus scrofa*. P1: first premolar, P2: second premolar, P3: third premolar, dP4: deciduous fourth premolar, M1: first molar, M2: second molar, M3: third molar.

A single mouse first molar tooth germ cultured *in vitro* had a cervical tongue in almost every intercuspal region (Fig. 3f). This result strongly indicates that there is a positive relationship between cusp number and the number of cervical tongues and roots. However, this simple positive relationship was not enough for us to explain the differences between crown and root numbers, especially in cases where the root number is greater than the cusp number. Many premolars of the mandible and maxilla of carnivores have one cusp and two roots, and the mandibular first molar of *Mustela sibirica* has three cusps and four roots. To explain this interesting phenomenon, we estimated the maximum number of both cervical tongues and roots in mammalian teeth according to a variety of cusp patterns and evaluated whether the root number of these teeth is greater than the cusp number by using simple formulas (Figs 4a and S3a–g). These formulas can be used for the prediction of the maximum number of cervical tongues and roots when the total cusp number is same or one less than the product of the cusp number in a column and the cusp number in a row. The number of roots becomes the maximum if all the estimated cervical tongues meet in one place of the tooth (Fig. S3a–g). This formula is unsuitable for a unicuspid tooth with two roots.

**Figure 5:**
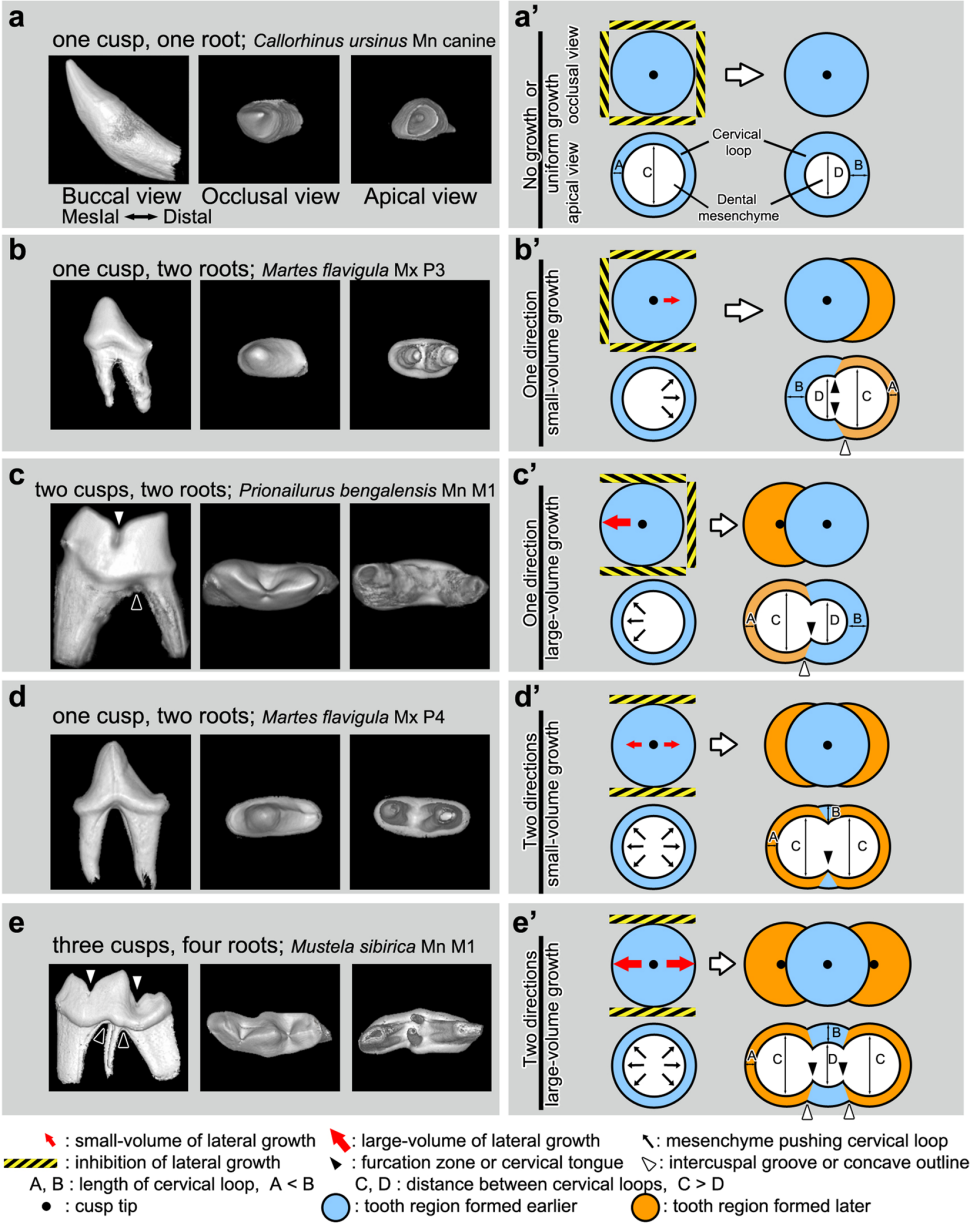
Schemes of cervical tongue patterning according to the lateral growth. **(a**,**a’)** In a unicuspid tooth, the lateral growth is blocked because the inhibition is in all axial direction. The cervical loop elongates uniformly toward the center of tooth germ, cervical tongue is not formed. **(b**,**b’**) In a unicupid tooth with one-directional lateral growth, dental mesenchyme in the inhibition-free region pushes the cervical loop out. The length of cervical loop in laterally growing region is shorter than that of cervical loop in lateral growth inhibited region. The further grown cervical loops form two cervical tongues. **(c**,**c’**) Bicuspid tooth can form two cervical tongues. The longer distal cusp compared to the mesial cusp indicates that the distal cusp is the early-formed cusp of the tooth. When the lateral growth occurs in the mesial side only, the lateral growth pushes the mesial cervical loop out. Notice that the position of root furcation (black arrowhead in **c**) is horizontally located between the tip of early-formed cusp and intercuspal groove (white arrowhead in **c**). This phenomenon is observed in the schematic diagram as well; the estimated cervical tongues (black arrowhead in **c’**) are more closely located between the tip of early-formed cusp and the intercuspal region (white arrowhead in **c’**). **(d**,**d’)** Two cervical tongues are formed in unicuspid tooth with bidirectional growth making two roots. **(e**,**e’)** Bidirectional growth in large-volume can form mesial and distal cusp additional to the tallest central cusp (**e**). Four cervical tongues can be formed at the boundaries between laterally growing regions and the inhibited regions. The furcation zone and cervical tongues (black arrowheads in **e** and **e’**) are located close to the central cusp from intercuspal grooves and intercuspal region (white arrowheads in **e** and **e’**).

Two linear and one broken-line graphs were drawn for the estimated maximum number of roots according to the number of cusps in teeth with one, two or three columns of cusps. In each graph for cusp columns, the maximum root number was proportional to the cusp number. A linear graph in teeth, where the number of cusp columns is one, shows that the maximum estimated root number exceeded the total cusp number; 4, 6, and 8 roots were estimated as the maximum number of roots in teeth with 3, 4, and 5 cusps, respectively. In teeth, where the number of cusp columns was two, the maximum estimated number of roots was exactly coincident with the total cusp number. In cases where both cusp column number and cusp row number are larger than two, the maximum root number was estimated to be smaller than the total cusp number. Our formulas indicate that the root number of a tooth can be greater than the cusp number only when the cusps are aligned in a column or in a row. The case in which the root number is larger than the cusp number can be observed in real teeth. The mandibular first molar of *Mustela sibirica* has three cusps and four roots, which is consistent with the estimated maximum root number in teeth with three cusps in a column (Figs 4b and 5e and S3a). The maxillary third and fourth premolars of *Martes flavigula* and *Mustela sibirica* have one cusp and two roots (Figs 4b and 5b,d). However, this formula was not able to explain the formation of two roots in a tooth with one cusp as described above.

There was no case where the actual number of roots exceeded the maximum estimated number of roots. The actual number of roots in various mammalian premolars and molars was the same or less than the estimated maximum number of roots regardless of the number of cusp columns (Figs 4b,c and S3b,d–g).

## Discussion

In previous studies regarding tooth development, a great degree of interest has focused on crown morphogenesis. However, root morphogenesis is poorly understood.

### Cervical tongues in the determination of root pattern

Although not much research has addressed root morphogenesis, cervical tongues have been considered to play an important role in tooth root pattern formation. It is known that the number and location of cervical tongues determine the number of tooth roots and the furcation zones^10,11,13–15^.

In the present comparative study between mouse and rat first molars in the mandible and maxilla, more roots and cervical tongues were observed in rats than in mice. These results confirm that the root number is reflected by the number of cervical tongues. Additionally, the discrete regions formed by the contact of cervical tongues correlate with the future furcation zone. These results confirm that the pattern of cervical tongues is essential in the determination of root pattern. Additionally, in our study, cervical tongues appeared from the bell stage, which is developmentally much earlier than the root forming stage in mouse in which HERS is formed. Therefore, it was confirmed that cervical tongues represent the tongue-like epithelium extending from the cervical loop rather than HERS.

### Relationship between crown pattern and cervical tongue pattern

There is a wide variation in body and molar size even in the same genus in the family Muridae^16–18^. Furthermore, molars in spiny rat (*Maxomys*) and black or brown rat (*Rattus*) are similar in crown size but completely different in root number^5,18^. *Maxomys* and *Rattus* have three and five roots in the maxillary first molar, respectively, and two and four roots in the mandibular first molar^5,18^. Therefore, the crown size difference between first molars of mouse and rat was not taken into account in the present study. In the present study, cervical tongues were observed in the concave intercuspal region of mouse and rat first molars. In intercuspal regions where only the rat has well-developed cervical tongues, the values of intercuspal distance and axial outline concavity were greater in rats than in mice. These results clearly suggest that intercuspal distance and axial outline are the important crown morphological features that affect the pattern formation of cervical tongues.

A prominent cervical tongue was observed in the intercuspal region between the lingual anterocone and anterostyle in rat maxillary first molars showing the shortest intercuspal distance but the highest concavity of the axial outline. There was a prominent cervical tongue in the intercuspal region between the entostyle and hypocone, where the axial outline was not concave. These results suggest that the intercuspal distance and axial outline should be considered as co-features of the crown pattern related to cervical tongue formation. A previous study suggested that the particular axial outline shape, such as triangular, square and rectangular, is related to the arrangement of roots by comparing the axial outline among maxillary teeth^8^. The present study is the first report to suggest that the concave intercuspal region is where cervical tongues form and that the concavity of the axial outline substantially affects the formation of cervical tongues and roots even in similar axial outline shapes.

### Cervical tongues in intercuspal regions as passive products of lateral growth in the cuspal region

Cervical tongues were not distinguishable from the neighboring cervical loop by epithelial cell proliferation at the bell stage, as most epithelial cells of the cervical loop were actively proliferating. Interestingly, the difference in cell proliferation was observed in the dental mesenchyme rather than in the dental epithelium. The number of proliferating mesenchymal cells was elevated in the lateral side of dental papilla in the cuspal region rather than in the center of the dental papilla. These proliferating dental mesenchymal cells may inhibit the invagination of the cervical loop or push the cervical loop laterally, depending on the developmental stage of the tooth germ. This hypothesis is consistent at the conceptual level with the recent report on the root itself from mouse PN3, which suggested that spatially regulated mesenchymal proliferation forms a physical barrier against the invagination of HERS during the elongation of roots^19^. The distance between buccal and lingual cervical loops in the cuspal area of all first molars increased from E16 to PN2 or PN4 and decreased from PN6. This result suggests that this condensed population of proliferating dental mesenchymal cells may push the dental epithelium and cervical loop laterally in the cuspal region of tooth germs at the bell stage, while the residual cervical loop in intercuspal regions that are not pushed away appears as a cervical tongue. This may signify that cervical tongue developing in the intercuspal region is a passive product of higher dental mesenchyme cell proliferation in the cuspal region.

### Inhibition of the lateral growth decreases the cervical tongue number

Compared to tooth germs *in vivo*, the axial outline of *in vitro* cultured single tooth germs was more convex in the cuspal area and concave in the intercuspal area. Tooth germs cultured *in vitro* had five cervical tongues in the concave intercuspal regions and the tips of all cervical tongues were sharp. When *in vivo*-like inhibition from surrounding tissue was created *in vitro*, the tooth germ between other tooth germs or glass barriers showed a flat axial outline where it contacted other tooth germs or glasses, and the length and shape of cervical tongues became short and blunt, respectively. These results suggest that the surrounding tissue physically inhibits the lateral growth of tooth germ, decreases the degree of both convexity and concavity in the axial outline and limits the number of cervical tongues in the tooth germs.

### Formula predicting the maximum number of roots

Cervical tongues were observed in most of the intercuspal regions of tooth germs cultured *in vitro*, which strongly suggests a positive relationship between the number of cusps and the number of cervical tongues. In the present study, we predicted the maximum number of cervical tongues and roots in various mammalian teeth according to their cusp patterns and evaluated whether the root number of these teeth could be greater than the cusp number. The predicted root number was larger than the cusp number in teeth where all cusps were arranged in a single column or row. This is the first report showing that the number of roots can exceed the number of cusps by using simple formulas.

The maximum number of roots was estimated through two steps in our formulas. In the first step of formula, the cervical tongue number was estimated according to the cusp pattern. In the second step of formula, the maximum root number was predicted from the cervical tongue number. To estimate the maximum number of cervical tongues and roots, the lateral growth inhibition was not considered in the first step, and it was assumed that all the cervical tongues meet in one place at the second step (Fig. S3). However, the lateral growth inhibition suppresses the formation of cervical tongues *in vivo*, and it is less likely that all cervical tongues meet in one place as the number of cervical tongues increases (Fig. S3c,g). The actual numbers of roots in various mammalian premolars and molars were the same or less than the estimated maximum number of roots. The difference between the actual root number and the maximum number may result from both the decreased number of cervical tongues caused by the lateral inhibition from surrounding tissues *in vivo* and a low probability that all cervical tongues meet in one place in multi-cusped teeth. Because of this tendency, the difference between the maximum estimated root number and the actual root number can be large as the cusp number increases.

Previously, by applying the cusp number only, it was impossible to identify a linear relationship between cusp and root number in both the mandible and maxilla. In the present study, by constructing each linear graph for the respective number of cusps in a column, we suggest that there is a positive relationship between cusp number and maximum root number. Especially in teeth where the number of cusp columns is one or two, there was a strict linear relationship between cusp number and maximum root number.

### Regulation of cusp and cervical tongue patterning by lateral growth during tooth development

In order to examine whether various cusp patterns and root patterns observed in actual mammalian teeth can be simulated, unicuspid teeth with two roots in particular, we tried to predict the various patterns of cusps and cervical tongues resulting from changes in lateral growth and the inhibition of lateral growth during sequential developmental stages of teeth in simple schemes.

The scheme was outlined on the basis of several simplified parameters. First, there is a circular rim of the cervical loop in the entire region where a cusp exists or lateral growth takes place. Second, as tooth germ develops, the circular rim size of the cervical loop corresponding to the distance between cervical loops decreases. Third, the circular rim size is smaller in the cusps that develop earlier than in later developing cusps. Fourth, the place in which two circular rims of cervical loops meet is considered the cervical tongue.

If lateral growth of a tooth germ is completely blocked by surrounding tissues or is uniformly present in the tooth germ, the cervical loop evenly grows during development; therefore, a cervical tongue may not be observed (Fig. 5a’). On the other hand, if lateral growth is present non-uniformly in a tooth germ, the lateral growth in certain cuspal regions may push the surrounding cervical loop outward, and then cervical tongues can appear in the intercuspal region (Fig. 5a’).

Unicuspid teeth typically have one or two roots (Fig. 5a). In unicuspid teeth with a round crown axial outline, such as canines and premolars of carnivores, a cervical tongue is not formed. As the cervical loop grows during tooth development, the length of the cervical loop may increase and the distance between cervical loops decrease (Fig. 5a’). In this case, the cervical loop elongates uniformly, and only one root may be formed.

Unicuspid teeth with a buccolingually flat axial outline, such as upper and lower premolars of carnivores, usually have two roots (Fig. 5b,d), which might have resulted from fusion between the two cervical tongues during development. Two cervical tongues can be formed in a unicuspid tooth by one or two directional lateral growth in small-volume (Fig. 5b’,d’). The inhibition of buccolingual growth in earlier-formed regions may induce the mesial and/or distal growth to form the new tooth region later. Since lateral growth in the mesenchyme of a new tooth region pushes the cervical loop mesially and/or distally, the length of the cervical loop and the distance between cervical loops may be shorter and longer, respectively, in the later-formed tooth region than the earlier-formed tooth region. This differential growth of the cervical loop might cause the formation of two cervical tongues. One directional lateral growth in large-volume may form a new cusp, two intercuspal regions and two cervical tongues (Fig. 5c,c’), while two directional lateral growth in large-volume may form three cusps, four cervical tongues and four roots (Figs 5e,e’ and S3a).

Variation in the appearance sequence of individual secondary enamel knots results in diverse shapes of teeth found across vertebrates^20^. The more cusps a tooth has, the more steps of cusp formation must be involved. In molars with two or more cusps, the width of the root under the tooth region formed earlier is smaller than that of the tooth region formed later (Fig. 5c,e). In other words, the location of the furcation zone does not match horizontally with the position of the intercuspal groove but is located close to the tip of the cusp formed earlier rather than that of the cusp formed later. Interestingly, this phenomenon was also observed in our schemes. The predicted cervical tongues in schemes were formed close to the tip of cusp formed earlier rather than those of cusps formed later (Fig. 5c’,e’). Because the point at which the two different sized circles meet was always located closer to the center of the smaller circle, the tip of cervical tongues was weighted horizontally towards the tip of the cusp formed earlier rather than being in line with the intercuspal groove. These findings suggest that our simple but novel mechanism in which the lateral growth of the dental mesenchyme pushes the cervical loop outward in the cuspal region and then the cervical tongue appears in the intercuspal region, has a high potential to be the actual mechanism of cervical tongue pattern formation.

Until now roots have been generally neglected from observation and there are few reports in root patterning defects in the genetically altered mice showing crown pattern changes. Studying the root phenotypes in the mutant mice may enhance the understanding the molecular mechanism of root patterning.

In summary, cervical tongue, which is a critical structure in root patterning, appeared from the bell stage of tooth development when crown morphogenesis is still in progress. The pattern formation of cervical tongues and roots was related to morphological features in the crown pattern. We suggested a simple mechanism in which lateral growth in the cuspal region increases the concavity and convexity of the axial outline and induces cervical tongue formation in intercuspal regions. The inhibition of lateral growth by surrounding tissues suppressed the cervical tongue formation, which confirms our mechanism. We presented formulas predicting the maximum number of cervical tongues and roots and showing the positive relationship between cusps, cervical tongues and roots. Furthermore, we demonstrated simple schemes to simulate the patterning of cusps, cervical tongues and roots and to show how a large number of roots can be formed in teeth with a small number of cusps.

## Materials and Methods

All experimental protocols were approved by the Yonsei University College of Dentistry, Intramural Animal Use and Care Committee. All experiments were performed in accordance with the guidelines and regulations of Yonsei University College of Dentistry, Intramural Animal Use and Care Committee.

### Whole-mount immunohistochemistry

Tooth germs were dissected out from ICR mice and SD rats at various stages. Tooth germs were fixed in methanol:DMSO (4:1) and bleached in methanol:DMSO:H_2_O_2_ (4:1:1). Samples were rehydrated and incubated in 1:200 diluted anti-E-cadherin antibody (R&D Systems). The color reaction was carried out by adding DAB chromogen solution from SuperPicTure Polymer Detection Kit (Thermo Fisher Scientific) to the tissue. The pattern of cervical tongues in the base of tooth germs was observed from apical view.

### *In situ* hybridization

Tissues were fixed overnight in 4% paraformaldehyde and hybridized with digoxigenin-labeled cRNA probes in hybridization buffer. Hybridization signals were detected by alkaline-phosphatase-conjugated anti-digoxigenin antibodies plus nitro blue tetrazolium chloride and 5-bromo-4-chloro-3-indolyl phosphate, toluidine salt substrate (Roche).

### Quantitative evaluation of cell proliferation

Slides with 6 µm paraffin-embedded sections were incubated in antibody against Ki67 (Thermo Fisher Scientific) and incubated with secondary antibody and streptavidin peroxidase. Results were visualized using a DAB substrate kit (Thermo Fisher Scientific). The cell number of dental papilla was counted in each five frontal serial sections using ImageJ. The counted area was delimited by 50 µm * 50 µm square in the center and the lateral sides of dental papilla. The Ki67-positive proliferation index was calculated as the percentage of Ki67-positive cells to the total number of cells.

### *In vitro* organ culture

First molar tooth germs were dissected out from E14 mice. Tooth germs were cultured in DMEM/F12 containing 10% FBS (GIBCO), a final concentration of 100 U/mL penicillin-streptomycin and 150 μg/ml ascorbic acid. The base of tooth germs were placed on the membrane downward and cultured *in vitro* (Fig. S2a). More than ten sets of tooth germs were cultured in solo, in triplet or with glass barriers, respectively, for 4 days *in vitro* as described previously^21,22^. Thickness of glass barriers was about 500 µm, and two glasses were placed about 400 µm apart from each other, which corresponds to the buccolingual width of the central tooth germ in triplet culture after 4 days. A single tooth germ was inserted into the gap between these glass barriers (Fig. S2a).

### Three-dimensional (3D) reconstruction using computed tomography

Computed tomographic images were obtained by scanning the dry skull using Skyscan 1076 (Bruker) and Alphard 3030 (Asahi Roentgen). The data were then digitalized using a frame grabber and the resulting images were transmitted to a computer with topographic reconstruction software Rapidform2006 (INUS Technology); then, premolars and molars were segmented out.

### Crown morphological analysis

Maxillary and mandibular first molars were extracted around eruption of first molars at PN14 in mice (n = 5) and PN21 in rats (n = 5) to prevent the crown shape from changing due to occlusal wear and were three dimensionally reconstructed.

For the distance between cusps, reference points were made at the highest tip of each cusp, and then a straight line connecting two neighboring tips was measured. In order to adjust the measurements by the relative size of a tooth, the measurements were corrected by dividing each measurement by the mesiodistal length of each tooth.

For axial outline analysis, the two-dimensional outline from an occlusal view was quantified using the Radial Fourier method as described previously^23,24^. For the degree of concavity, tangent lines touching two convex outlines next to an intercuspal region were created in each tooth, and the area between the outline and the tangent line was scaled to the total area of the outline in order to compensate for the size difference between mouse and rat. For each analysis, Student’s *t*-test was performed using SPSS 20.0 (IBM Corporation) and the level of statistical significance was *P* < 0.05.

### Formula for the prediction of the maximum number of cervical tongues and roots

As for the maximum number of cervical tongues, a cervical tongue was set to appear in every intercuspal region (Fig. 4a).

First, when the number of cusps in a respective column or row is the same, the total number of cusps is the product of the number of cusps in a column and the number of cusps in a row. As for the maximum number of roots, when all cervical tongues meet at one site, the root number will reach its maximum number, which is the same as the total number of cervical tongues. However, as the number of cervical tongue increases, it is less likely for all cervical tongues to meet at one site. Therefore, equations are as follows:

1 $$X=AB,$$

2 $$Y\le {2}(A+B-{2}),$$

3 $$Z\le Y,$$

where *A*, *B, X*, *Y* and *Z* are numbers of cusp columns, cusp rows, total cusps, cervical tongues and roots, respectively. The required conditions are *A* ≥ *1* and *B* ≥ *1*. Exceptionally, the root number becomes one (*Z* = *1*) when a cervical tongue does not appear (*Y* = *0*).

Secondly, when the cusp number is one less than the product of the number of cusps in a column and number of cusps in a row, the equations for the number of cusps, the number of cervical tongues, and the number of roots are:

4 $$X=AB-{1},$$

5 $$Y\le {2}(A+B-{2})-{1},$$

6 $$Z\le Y,$$

where *A*, *B, X*, *Y* and *Z* are numbers of cusp columns, cusp rows, total cusps, cervical tongues and roots, respectively. The required conditions are *A* ≥ *2* and *B* ≥ *2*.

## Electronic supplementary material

Supplementary information
