# Supplementary material for: Regulation of root patterns in mammalian teeth
Source: Sci Rep. 2017 Oct 5;7:12714. doi: 10.1038/s41598-017-12745-1 (PMC5629201; doi:10.1038/s41598-017-12745-1)
Supplement: Supplementary file 1 — Supplementary information [file 41598_2017_12745_MOESM1_ESM.doc]

**Regulation of root patterns in mammalian teeth**

Hyejin Seo^1,2^, Jinsun Kim^1,2^, Jae Joon Hwang^3^, Ho-Gul Jeong^3^, Sang-Sun Han^3^, Wonse Park^4^, Kanghyun Ryu^5^, Hong Seomun^6^, Jae-Young Kim^7^, Eui-Sic Cho^8^, Joo-Cheol Park^9^, Kyung-Seok Hu^1,2^, Hee-Jin Kim^1,2^, Dong-Hyun Kim^4,*^ and Sung-Won Cho^1,*^

**Supplementary Information**

**
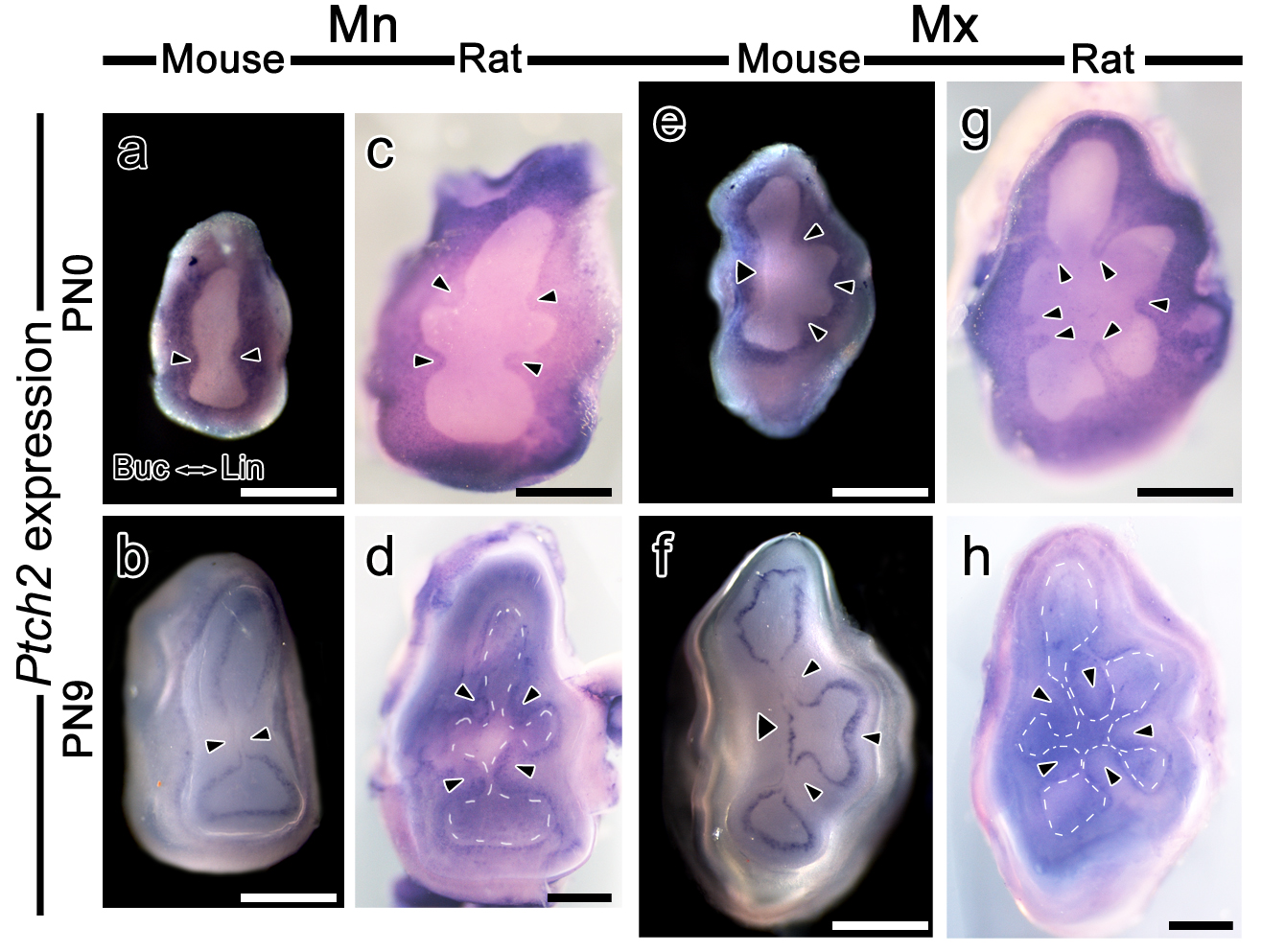
**

**Fig. S1.Expression pattern of *Ptch2* in the margin of cervical loop. (a―h)** Expression pattern of *Patched 2* (*Ptch2*) in mandibular (Mn) and maxillary (Mx) first molars at postnatal (PN) 0 and PN9 in mouse and rat from apical view visualizes the margin of cervical loop and the shape of cervical tongues (arrows). The discontinuous lines illustrate the margin of cervical loop (d and h). Buc: buccal, Lin: lingual , Scale bars: 500 µm.


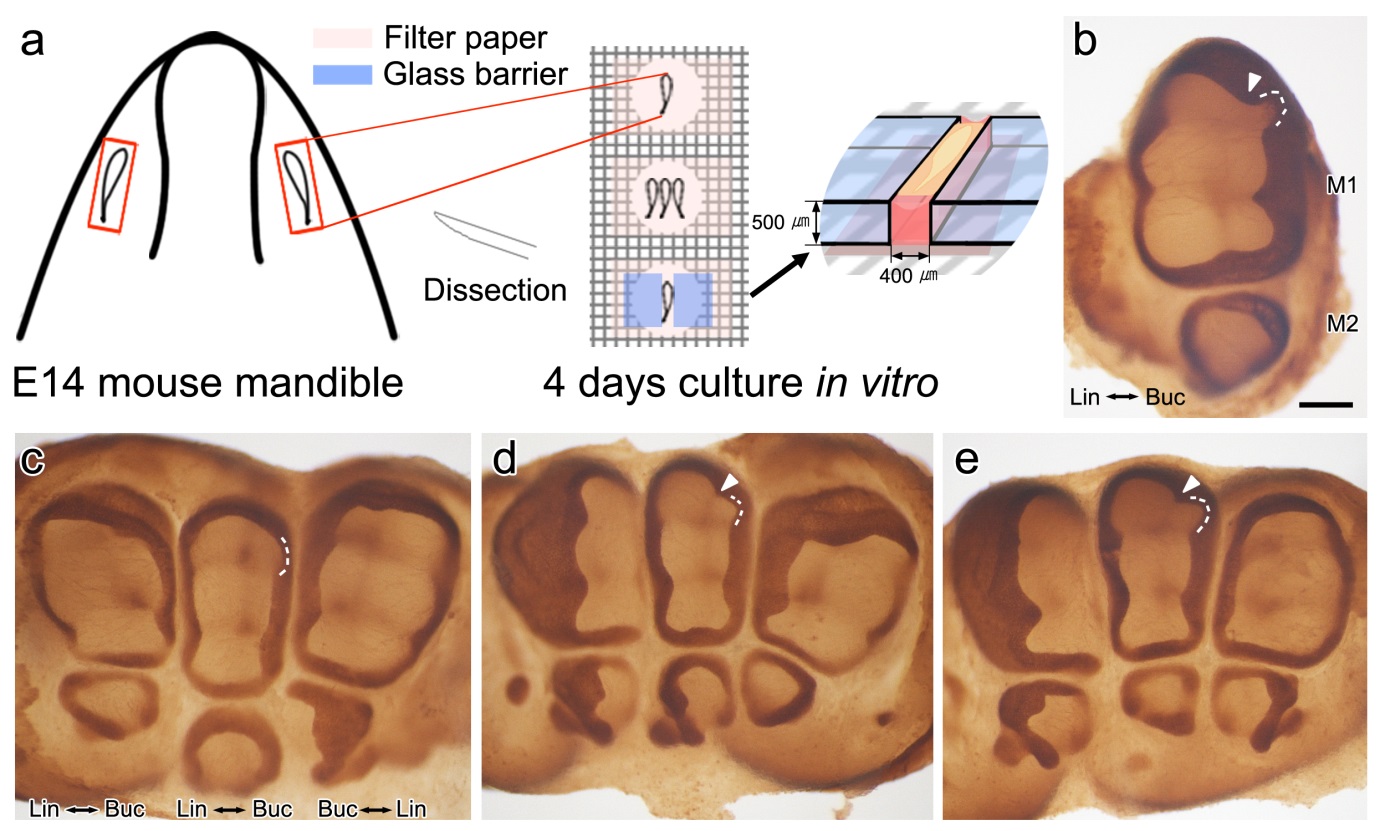


**Fig. S2.Alterations in cervical tongue patterns in tooth germs cultured *in vitro* by lateral inhibition. (a)** Schematic diagram depicting how lateral growth was manipulated around tooth germs which were dissected out from E14 mouse mandible. Tooth germs were cultured for 4 days *in vitro*. **(b)** Tooth germs cultured in solo show prominent a cervical tongue in between buccal and lingual anteroconids (white arrowhead). **(c)** In tooth germs cultured side by side, no cervical tongue is observed between the buccal and lingual anteroconids, though the mesial side of the central tooth germ does not contact with other tooth germs. **(d, e)** The less the buccal anteroconid (dashed white line) contact with adjacent tooth germ, the longer and sharper the cervical tongue developed between the buccal and lingual anteroconids. Scale bar: 200 µm.


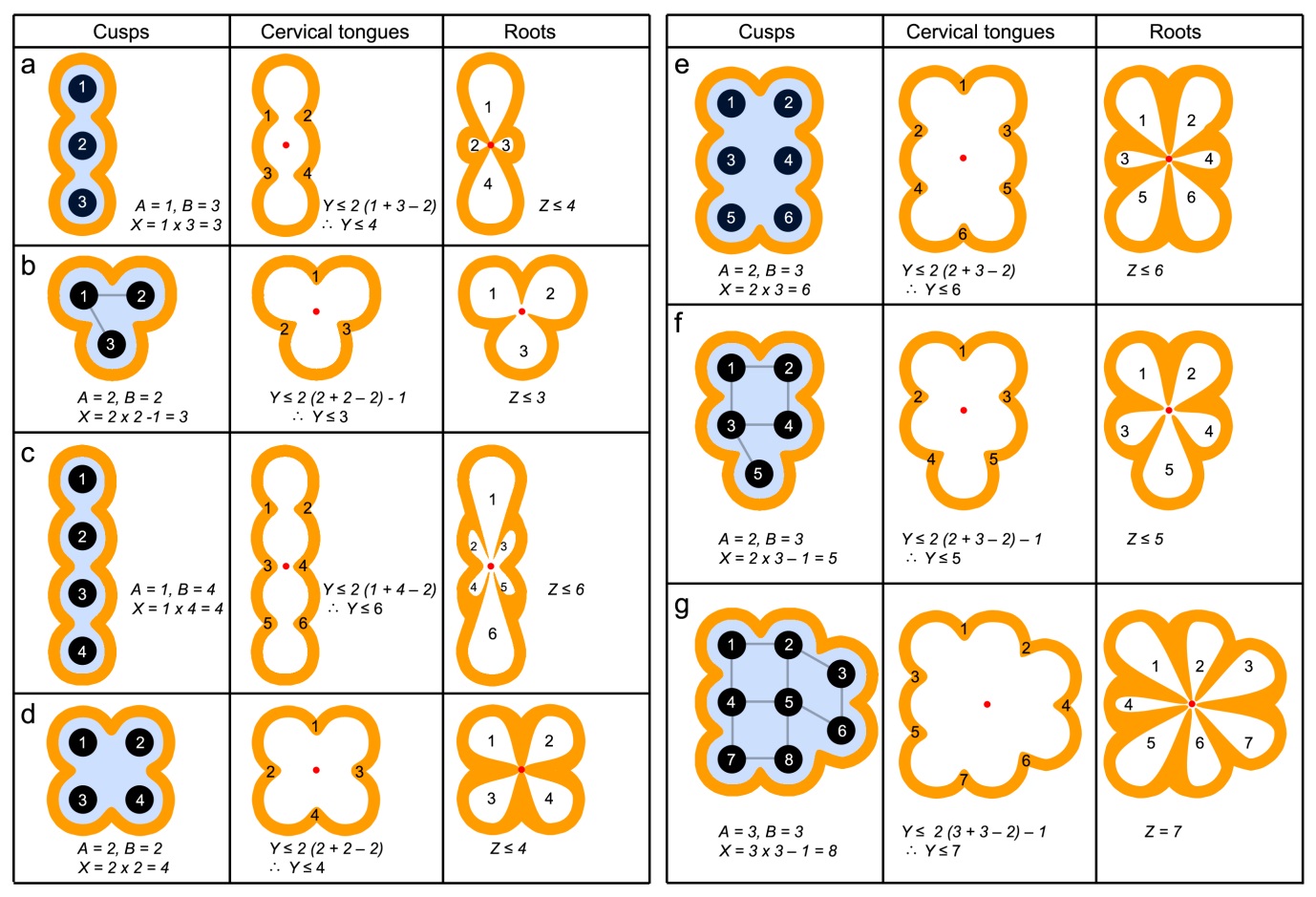


**Fig. S3.Alterations in patterns of cervical tongues and roots depending on cusp patterns. (a, c, d, e)** The expected patterns of cervical tongues and root patterns when the number of cusps in respective column or row is constant. If all the estimated cervical tongues meet in one place (small red dot in the center), the tooth will has the maximum number of roots. **(b, f, g)** The estimated patterns of cervical tongues and roots when cusp number is one less than the product of number of cusps in a column and number of cusps in a row. Notice that if the cusp arrangement is different among teeth, even though the cusp number is same, the number and arrangement of cervical tongues and roots becomes different.

*A, B, X, Y* and *Z* are numbers of cusp columns, cusp rows, total cusps, cervical tongues and roots, respectively.
